# Visualizing Early Splenic Memory CD8^+^ T Cells Reactivation against Intracellular Bacteria in the Mouse

**DOI:** 10.1371/journal.pone.0011524

**Published:** 2010-07-12

**Authors:** Marc Bajénoff, Emilie Narni-Mancinelli, Frédéric Brau, Grégoire Lauvau

**Affiliations:** 1 Institut National de la Santé et de la Recherche Médicale Unité 924, Groupe Avenir, Valbonne, France; 2 Université de Nice-Sophia Antipolis, Nice, France; 3 Centre d'Immunologie de Marseille-Luminy, INSERM, UMR-S 631, Marseille, France; 4 CNRS, UMR 6102, Marseille, France; 5 Université de la Méditerranée, UM 631, Marseille, France; 6 CNRS-UMR6097, IPMC, Valbonne, France; 7 Department of Microbiology and Immunology, Albert Einstein College of Medicine, New York, New York, United States of America; University of Toronto, Canada

## Abstract

Memory CD8^+^ T cells represent an important effector arm of the immune response in maintaining long-lived protective immunity against viruses and some intracellular bacteria such as *Listeria monocytogenes* (*L.m*). Memory CD8^+^ T cells are endowed with enhanced antimicrobial effector functions that perfectly tail them to rapidly eradicate invading pathogens. It is largely accepted that these functions are sufficient to explain how memory CD8^+^ T cells can mediate rapid protection. However, it is important to point out that such improved functional features would be useless if memory cells were unable to rapidly find the pathogen loaded/infected cells within the infected organ. Growing evidences suggest that the anatomy of secondary lymphoid organs (SLOs) fosters the cellular interactions required to initiate naive adaptive immune responses. However, very little is known on how the SLOs structures regulate memory immune responses. Using *Listeria monocytogenes* (*L.m*) as a murine infection model and imaging techniques, we have investigated if and how the architecture of the spleen plays a role in the reactivation of memory CD8^+^ T cells and the subsequent control of *L.m* growth. We observed that in the mouse, memory CD8^+^ T cells start to control *L.m* burden 6 hours after the challenge infection. At this very early time point, *L.m*-specific and non-specific memory CD8^+^ T cells localize in the splenic red pulp and form clusters around *L.m* infected cells while naïve CD8^+^ T cells remain in the white pulp. Within these clusters that only last few hours, memory CD8^+^ T produce inflammatory cytokines such as IFN-γ and CCL3 nearby infected myeloid cells known to be crucial for *L.m* killing. Altogether, we describe how memory CD8^+^ T cells trafficking properties and the splenic micro-anatomy conjugate to create a spatio-temporal window during which memory CD8^+^ T cells provide a local response by secreting effector molecules around infected cells.

## Introduction

The protective immune response against pathogenic microorganisms involves two components: a rapid, antigen non-specific innate response and a delayed, acquired response specific for the antigens (Ags) displayed by invading microbes. The efficacy of these responses relies on multiple effector functions such as the secretion of antimicrobial molecules and the direct killing of infected cells by effector lymphocytes [1]. However, these mechanisms are only the final steps of a long cascade of events that began several days before. Naïve T lymphocytes endlessly patrol the T cell zones of all secondary lymphoid organs (SLOs) [2], [3]. Upon an infection, homing to the relevant infected SLO represents the first challenge for rare Ag-specific T cells. In order to be primed, lymphocytes further need to find the Ag-loaded cells amongst millions of other SLO wandering cells. Growing evidences suggest that the structure of SLOs plays an active role in this search [4]. For instance, Ag-loaded DCs derived from inflamed tissues migrate via lymphatics to the draining LN where they settle close to High Endothelial Venules (HEVs), forming a “gauntlet” of Ag through which blood incoming cells will have to pass upon their exit from the HEVs [5]. Meanwhile, draining LNs massively recruit blood circulating naïve T cells via their inflamed HEVs, increasing the efficacy of this screening process [6], [7]. While the ability of a lymphocyte to visit all LNs is critical for a thorough monitoring of potential infections, it can also be viewed as distracting the cell from where it is mostly needed upon infection namely the SLO draining the infected site. This notion is crucial since lymphocytes wander approximately 12 hours in a given SLO before they can return to the blood circulation and get another opportunity to home to the SLO draining this inflamed tissue [3].

In this context, the rapidity and efficacy of memory cells to control secondary infections are puzzling. Memory CD8^+^ T cells are endowed with enhanced antimicrobial effector functions and properties including increased lifespan and higher division rate that perfectly tail them to rapidly eradicate invading pathogens [8]. However, such functional features would be useless if memory cells were unable to rapidly find the pathogen loaded/infected cells. Memory CD8^+^ T cells represent an important effector arm of the immune response in maintaining long-lived protective immunity against intracellular pathogens such as viruses or some intracellular bacteria [9]. Amongst these intracellular pathogens, the bacterium *Listeria monocytogenes (L.m)* has been widely used as an infection model to study memory CD8 responses. Upon recall infection, *L.m*-specific memory CD8^+^ T cells are able to control a dose of bacteria otherwise lethal for non-immunized animals. This rapid curving of bacterial growth occurs within a day or less, questioning how these cells locate and induce pathogen killing in such a small amount of time [10].

Memory T cells can be divided in two subsets based on the expression of the LN homing receptors CD62L and CCR7. Central memory T (T_CM_) cells are CD62L^+^ CCR7^+^ and preferentially localize to the T cell zones of SLOs whereas effector memory T (T_EM_) are CD62L^−^ CCR7^−^ and preferentially localize to peripheral tissues [11]. The majority of the memory CD8^+^ T cells generated following *L.m* infection belongs to the T_EM_ subset and therefore poorly colonize the T cell zones of SLOs [12], [13]. However, it has been suggested that memory CD8^+^ T cells reactivation is promoted by a cognate interaction with *L.m*-loaded CD11c^+^ cells that are known to migrate within a few hours to the splenic T cell zone, a region where T_EM_ poorly home [14], [15], [16]. In light of these observations, we addressed when and where memory CD8^+^ T cells are re-activated and provide their rapid effector functions for early control of bacterial growth during a secondary infection.

## Results

### Memory CD8^+^ T cells control secondary *L. monocytogenes* growth within the first hours of infection

**Figure 1:**
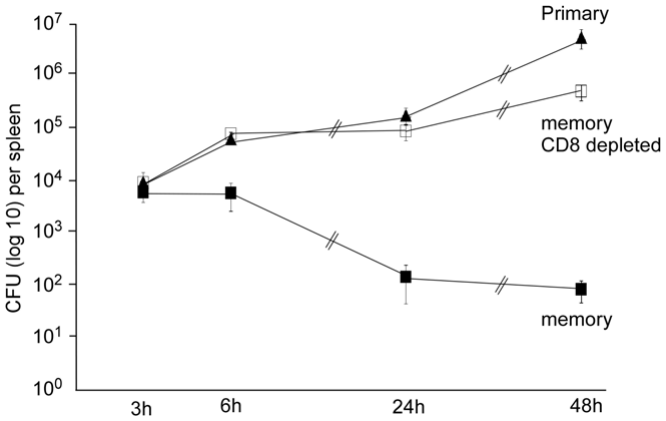
Memory CD8 T cells rapidly control secondary *L.m* infection. Mice (3 per group) were infected i.v with PBS or immunized with 0.1×LD_50_ Wt *L.m* bacteria. 30 days later, animals were injected or not with 100 µg of anti-CD8β depleting Ab i.p for three consecutive days and then challenged with 7×10^5^ Wt bacteria. Bacteria titers in the spleen were measured 3, 6, 24 and 48 hours later. Data are representative of 2 independent experiments.

To accurately determine when memory CD8^+^ T cells start controlling *L.m* growth during a secondary infection, we injected mice intravenously (i.v) with PBS or 0.1×LD_50_ Wt bacteria (10^4^) in order to induce a protective CD8 memory response. One month later, CD8^+^ T cells were eliminated or not with a depleting anti-CD8β treatment and mice were challenged with 10×LD_50_ Wt bacteria. Bacterial growth inside infected spleens was then monitored 3, 6, 24 and 48 hours following infection. As expected, immunized mice controlled the infection while CD8-depleted and PBS-injected animals exhibited at least 1000 fold more bacteria 24 hours after the challenge infection (Figure 1). Most interestingly, the control of *L.m* growth by memory CD8^+^ T cells was already visible after 6 hours, suggesting that memory cells not only had seen their cognate antigen at that time but had also started to kill bacteria inside infected spleens.

### *L.m* specific memory CD8^+^ T cells are mostly found inside the splenic red pulp

**Figure 2:**
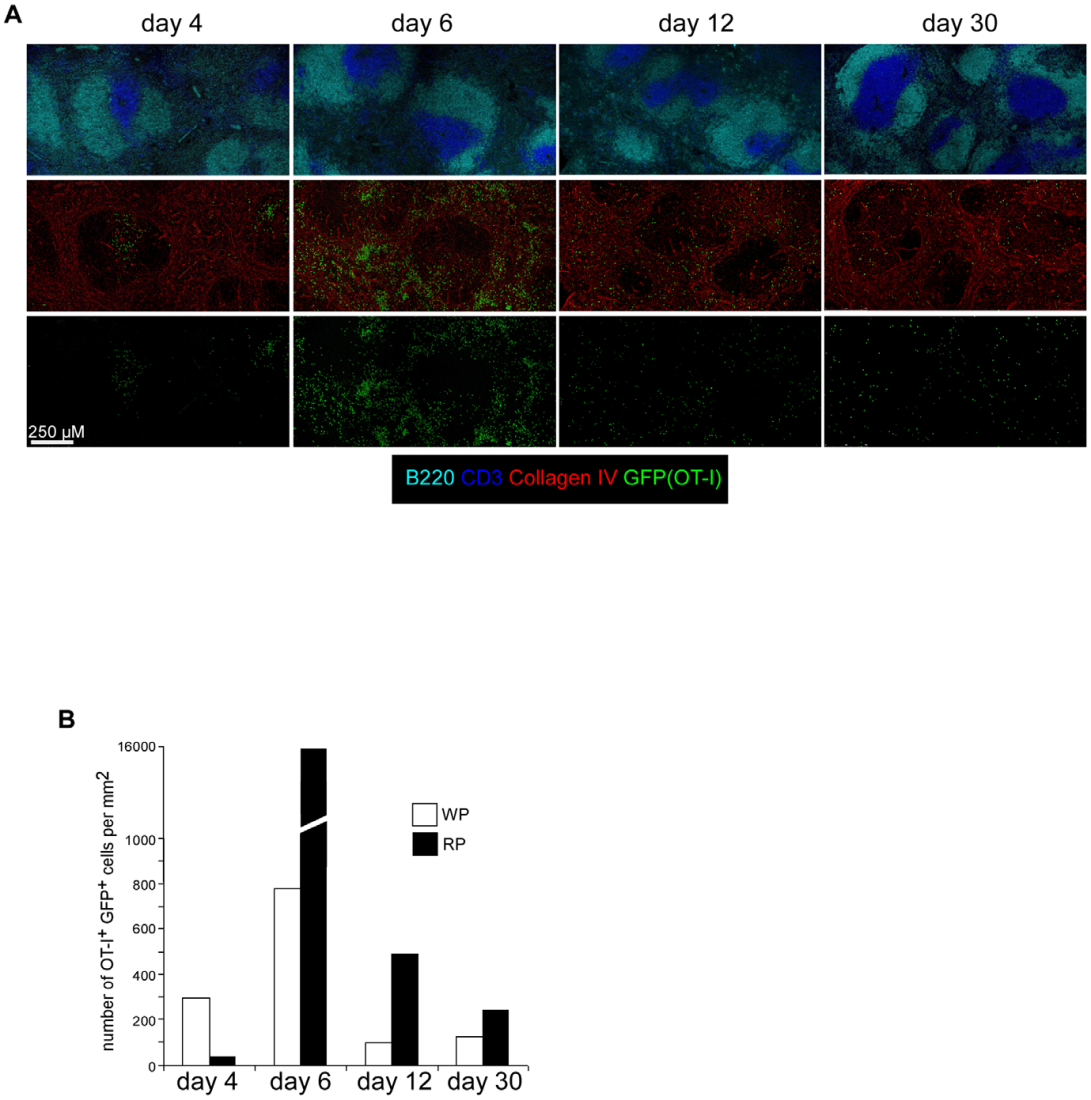
Within the spleen, *L.m* specific memory CD8 T^+^ cells predominantly reside in the Red Pulp. (A, B) 200 naïve GFP^+^ OT-I cells were transferred in C57BL/6 mice. One day after, recipient mice were injected i.v. with 0.1×LD_50_ Wt *L.m*-OVA. Spleens were harvested 4, 6, 12 and 30 days later, sectioned, stained for B220, CD3, collagen IV expression and analyzed by confocal microscopy. (B) The surfaces of WP and RP zones were delineated based on collagen IV expression and summed up. The densities of memory GFP^+^ OT-I cells per mm2 of each region were calculated and displayed. Data are representative of 3 independent experiments.

While naïve T cells traffic to the T cell zones of the SLOs, *L.m*-specific memory T cells have been shown to patrol the splenic RP and WP [17]. However, studies that visualized the behaviour of memory CD8 T cells were done either using non-physiologic numbers of naive T cell precursors and/or using approximate landmarks such as T, B or MOMA-1 stainings [17], [18]. Fibroblastic Reticular Cells (FRCs) residing in the T cell zones of SLOs dictate the migration and define the territory of naïve T lymphocytes in these organs [19]. As FRCs trespass in the B cell follicles, they still support the migration of T cells in the follicles, explaining the presence of sparse T cells adjacent to reticular fibers in these areas. Most importantly, the collagen IV- and ERTR-7-expressing reticular fibers they ensheat are reliable markers to accurately localize them on tissue sections [20]. For these reasons, we decided to use reticular fiber-specific markers to define the exact location of memory CD8^+^ T cells generated from a physiological number of naive CD8^+^ T cells precursors specific for a *L.m*-expressed antigen. Since a normal mouse possesses ∼200 T cells of a given specificity directed against a dominant epitope [21], [22], [23], [24] and CD8^+^ T cells frequencies affects the kinetics of their expansion and differentiation into effector and memory cells [25], we adoptively transferred 200 GFP^+^ OVA-specific OT-I CD8^+^ T cells (OT-I) in recipient mice that were injected i.v the day after with 0.1×LD_50_ Wt- or Wt-OVA expressing bacteria. Spleens were sectioned 4, 6, 12 and 30 days later and stained for B220, CD3, collagen IV expression (Figure 2A). As expected, we were unable to detect any of the 200 OT-I cells in mice immunized with Wt bacteria (data not shown). In Wt *L.m*-OVA infected animals, OT-I cells became detectable 4 days after injection and were mainly localized in the WP as previously observed [17]. At day 6, during their peak of proliferation, OT-I cells had massively expanded and colonized the RP where they predominantly remained during the contraction phase (day 12) and until day 30. In order to quantify these observations, we calculated the densities of OT-I cells per mm^2^ of RP and WP at these different time points (Figure 2B). Data reveal that the density of memory OT-I cells in the RP was two times higher than in the WP, indicating that memory OT-I cells predominantly colonize the RP of the spleen, correlating with the observation that 2/3 of the OT-I isolated from the same spleen belong to the T_EM_ subset (CD62L^low^ CCR7^low^, not shown).

**Figure 3:**
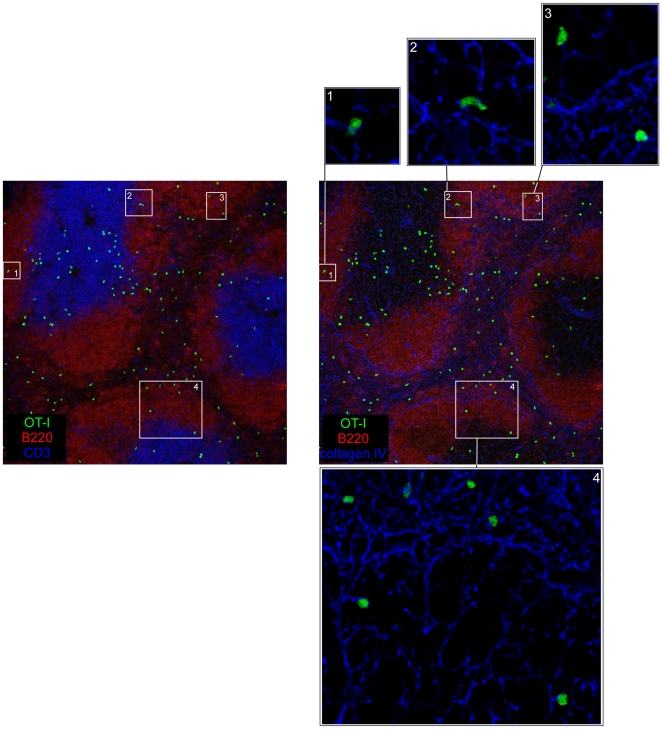
*L.m*-specific memory CD8^+^ T cells present in follicles are adjacent to reticular fibers. 200 naïve GFP^+^ OT-I cells were transferred in C57BL/6 mice that were injected i.v. the following day with 0.1×LD_50_ Wt *L.m*-OVA. Spleens were harvested 30 days later, sectioned, stained for B220, CD3, collagen IV expression and analyzed by confocal microscopy. Inserts show enlargements of B220^+^ areas containing OT-I memory cells. Data are representative of 2 different experiments.

Because memory OT-I cells have been reported to home to B cell follicles, we focused on OT-I cells that were present in these zones [17]. We observed that follicular OT-I cells were in contact with adjacent collagen IV reticular fibers, suggesting that at least some memory CD8^+^ T cells present in follicles are indeed patrolling “T cell zone embassies” created by residual FRCs (Figure 3). Collectively, our results suggest that the majority of *L.m*-specific memory CD8^+^ T cells remains in the RP on steady state.

### *L.m*-specific memory CD8^+^ T cells are continuously patrolling the blood system

**Figure 4:**
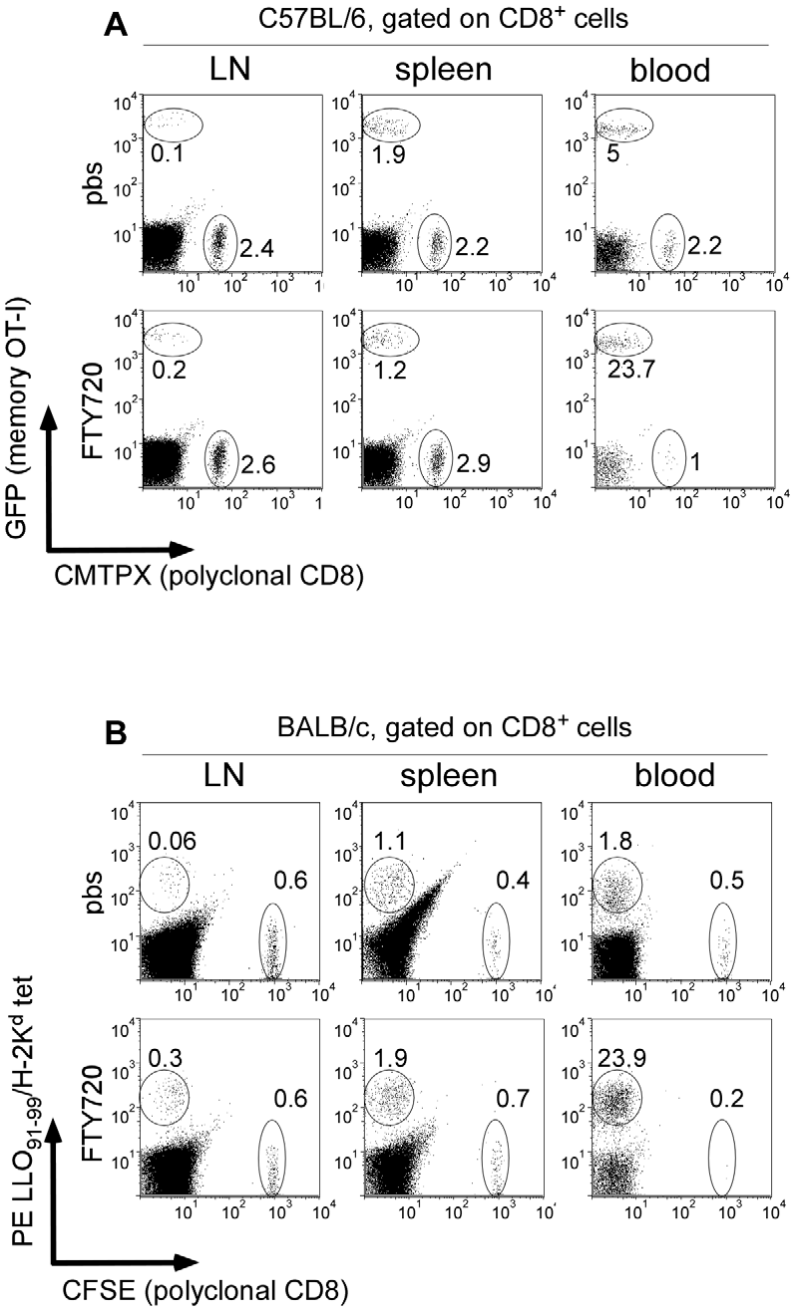
*L.m* specific memory CD8^+^ T cells preferentially traffic in the bloodstream. (A) 200 naïve GFP^+^ OT-I cells were transferred in C57BL/6 mice. One day later, recipient mice were injected i.v. with 0.1×LD_50_ Wt *L.m*-OVA. Thirty days after this immunization, recipient mice were injected i.v with 3×10^6^ naïve CMTPX-labelled polyclonal CD8^+^ T cells. The following day, mice were injected i.p with PBS or 100 µg of FTY720. Animals were killed 24 hours later and their LNs, spleen and blood harvested, stained for CD8 expression and analyzed by flow cytometry. Numbers indicate the percentages of GFP^+^ OT-I memory cells and CMTPX naive polyclonal CD8^+^ T cells among total CD8^+^ T cells. (B) Wt BALB/c mice were injected i.v with 0.1×LD_50_ Wt *L.m* and further treated as in (A) with the exception that polyclonal naive CD8^+^ T cells were labelled with CFSE and that *L.m* specific endogenous memory CD8^+^ T cells were identified as LLO_91_–_99_/H-2K^d^ tetramers^+^ CD8^+^ cells. Data are representative of 3 independent experiments.

The RP filters the blood content and can be assimilated to a blood-filled sponge rather than a truly organised tissue like the WP [26]. Therefore, we hypothesized that the presence of memory OT-I cells in the splenic RP may solely be the result of their endless trafficking in the bloodstream. To test this hypothesis, we adoptively transferred 200 GFP^+^ OT-I cells in recipient mice that were injected i.v. 1 day later with 0.1×LD_50_ Wt or Wt-OVA-expressing bacteria. One month later, 3×10^6^ polyclonal naïve CD8^+^ T cells were labelled with a Red tracker (CMTPX) and injected i.v. in the mice in order to generate an internal control of naïve T cell trafficking pattern. LNs, spleens, and blood cells were stained for CD8 expression and analyzed by flow cytometry (Figure 4A, upper panel). While the naive cells represented a stable proportion of total CD8^+^ T cells in the 3 compartments, memory OT-I cells were poorly present in the LNs of primed animals but more abundant in their blood and spleens, consistent with their CD62L^low^ CCR7^low^ phenotype (not shown). Similar results were obtained when following the endogenous memory CD8 response generated against the naturally processed listeriolysin (LLO)-derived epitope LLO_91_–_99_ presented by H-2K^d^ using LLO_91_–_99_/H-2K^d^ tetramers (Figure 4B, upper panel). Neither OT-I nor LLO_91_–_99_/H-2K^d^ tet^+^ cells were detected in mice injected 30 days earlier with Wt bacteria or PBS respectively (not shown).

As memory OT-I cells were predominantly found in the blood compartment of infected animals, we hypothesized that, unlike naïve cells that continuously patrol SLOs, they may not be retained in SLOs upon treatment with the S1P agonist FTY720, a potent inhibitor of naïve lymphocyte egress from lymphoid organs [27]. To address this question, *L.m*-immunized mice were injected i.p with 100 µg of FTY720 and their spleens, LNs and blood were analyzed 24 hours later by flow cytometry (Figure 4A and 4B, lower panels). While -as expected- injection of FTY720 induced a reduction of the naive population from the blood of treated animals [27], OT-I and LLO_91_–_99_/H-2K^d^ tet^+^ CD8^+^ T cells were highly enriched in the bloodstream. These results indicate that memory CD8^+^ T cells are poorly sensitive to the S1P agonist, further confirming their peculiar trafficking pattern that may prompt them to be more easily mobilized upon secondary infection.

### *L.m* specific memory CD8^+^ T cells rapidly form clusters in the RP during a recall infection

**Figure 5:**
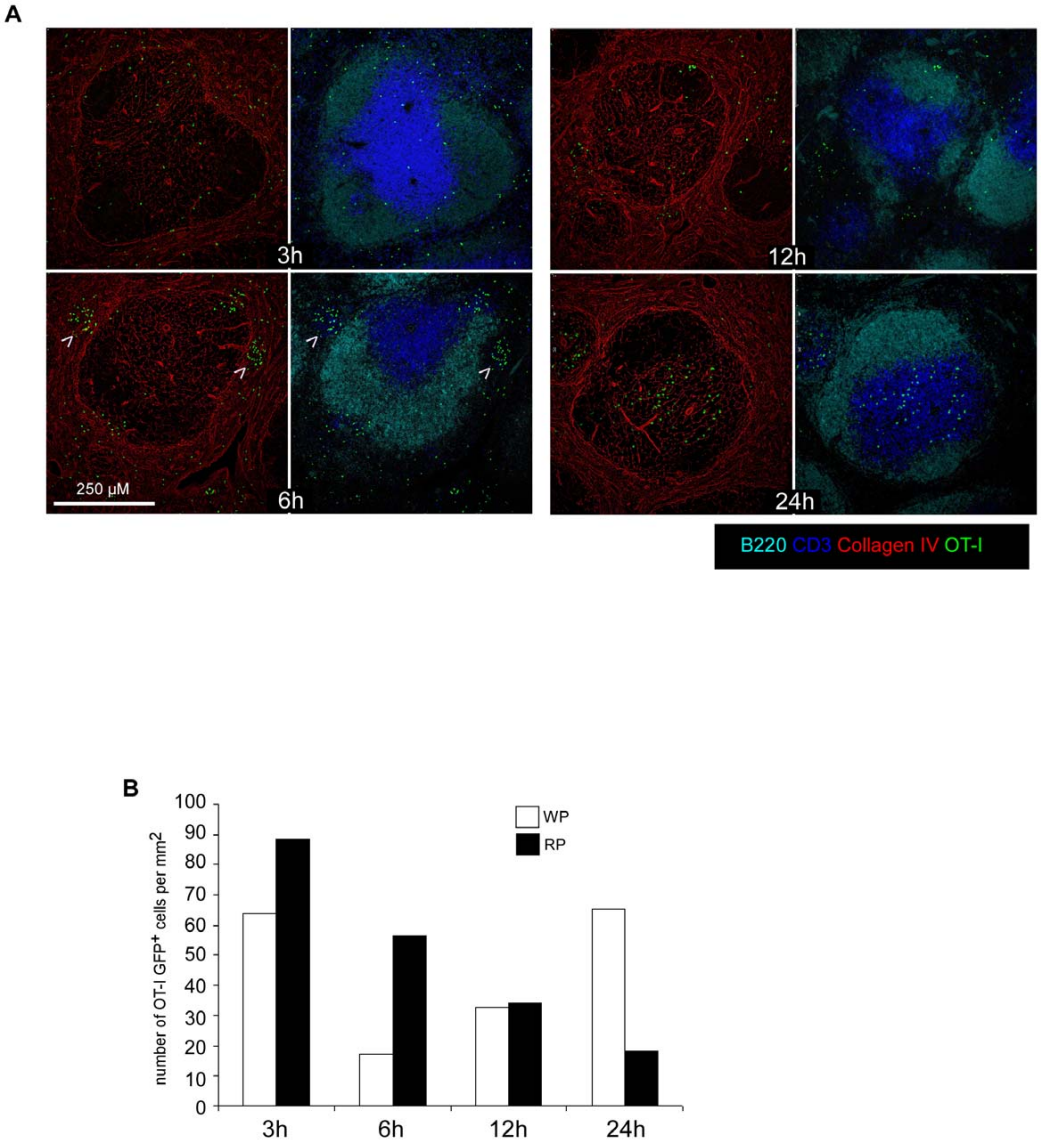
*L.m*-specific memory CD8^+^ T cells transiently form clusters in the Red Pulp of secondary infected animals. (A, B) 200 naïve GFP^+^ OT-I cells were transferred in C57BL/6 mice that were injected i.v. the following day with 0.1×LD_50_ Wt *L.m*-OVA. One month later, mice were injected i.v. with PBS or 10×LD_50_ Wt *L.m*-OVA. Spleens were harvested 3, 6, 12 and 24 hours later, sectioned, stained for B220, CD3, collagen IV expression and analyzed by confocal microscopy. Arrowheads indicate clusters of GFP^+^ OT-I memory cells 6 hours post re-infection. (B) The surfaces of WP and RP zones were delineated based on collagen IV expression and summed up. The densities of memory GFP^+^ OT-I cells per mm2 of each region were calculated and displayed. Data are representative of 3 independent experiments.

Because memory OT-I cells were predominantly found in the RP and in the bloodstream of infected mice, we reasoned that the early (6 hours) control of *L.m* growth should occur in the RP, before infected cells have reached the WP. To investigate these early events, 200 GFP^+^ OT-I cells were adoptively transferred in recipient mice that were injected 1 day later with 0.1×LD_50_ Wt *L.m*-OVA. After 30 days, animals were challenged with 10×LD_50_ Wt *L.m*-OVA. Spleens were harvested 3, 6, 12 and 24 hours later, sectioned and stained for B220, CD3, and collagen IV expression (Figure 5A). At 3 hours, memory OT-I cells were present in the WP and the RP of re-infected animals in the same proportions than untreated animals. Most interestingly, at 6 hours, we observed the formation of OT-I clusters in the RP of secondary infected animals (arrowheads). These clusters were transient since they were largely crumbled after 12 hours and completely dissolved at 24 hours, when OT-I cells had massively relocated to the T cell zone (Figure 5A). Quantification of the density of OT-I cells per mm^2^ of RP and WP confirmed this impression, indicating that a transient clustering of *L.m*-specific memory CD8^+^ T cells in the RP precedes their relocation to the WP (Figure 5B).

### *L.m*-specific memory ‘CD8 clusters’ concentrate immune cells expressing antimicrobial effector functions

**Figure 6:**
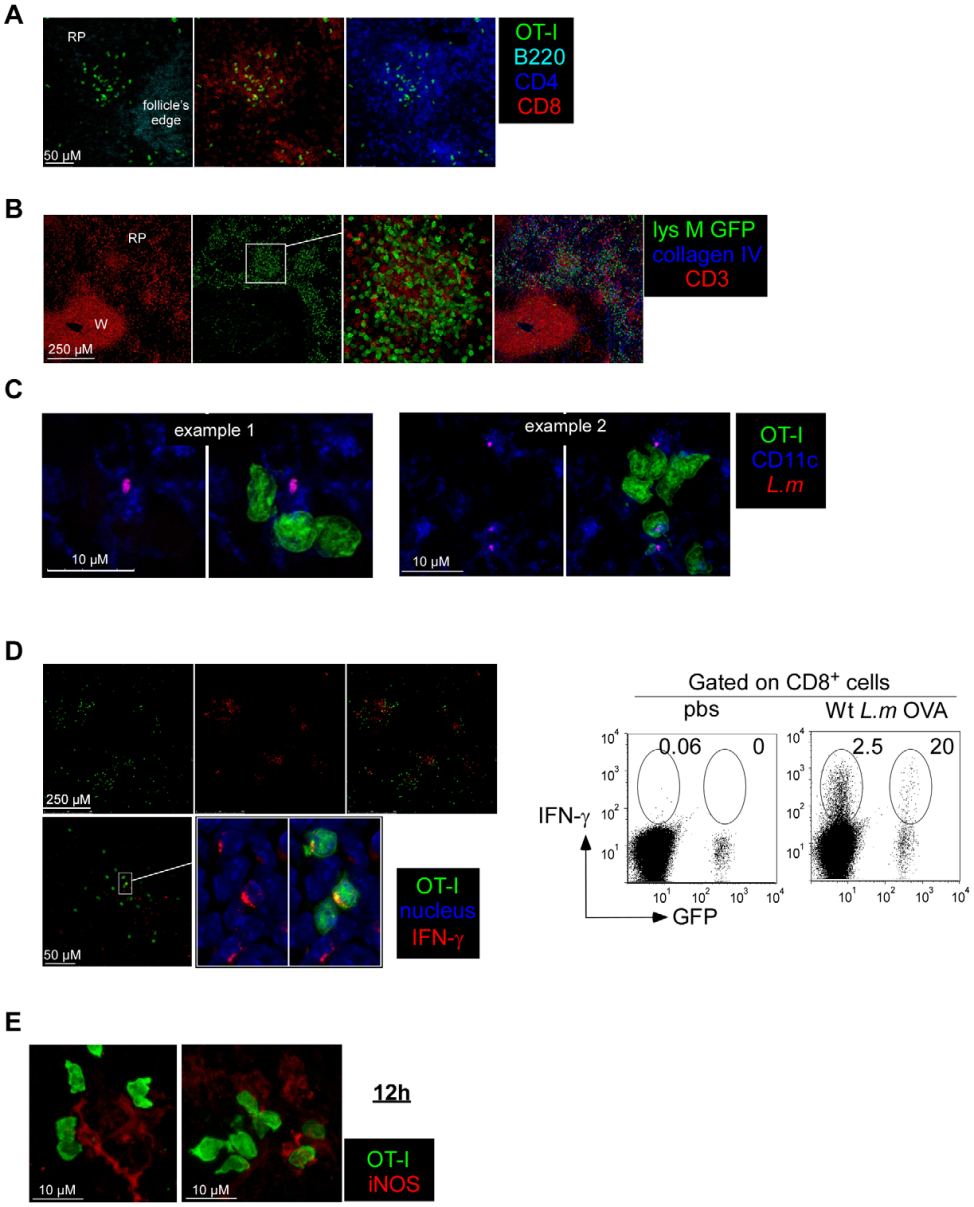
Memory ‘CD8 clusters’ concentrate anti-*listeria* effector cells. (A,C,D,E) 200 naïve GFP^+^ OT-I cells were transferred in C57BL/6 mice. One day after, recipient mice as well as naive GFP^+^ lys-M Tg mice (B) were injected i.v. with 0.1×LD_50_ Wt *L.m*-OVA. One month later, all mice were injected i.v. with 10×LD_50_ Wt *L.m*-OVA. Spleens were harvested 6 (A,B,C,D) or 12 hrs later (E), sectioned, stained with anti-B220, -CD4, -CD8 (A); anti-CD3, -collagen IV (B) anti-CD11c, -listeria (C); anti-IFN-γ, and hoescht (D), anti-iNOS (E) specific Abs and analyzed by confocal microscopy. In (D), IFN-γ secretion by endogenous CD8^+^ T cells and GFP^+^ OT-I memory cells was also assessed by flow cytometry. Data are representative of 3 independent experiments.

**Figure 7:**
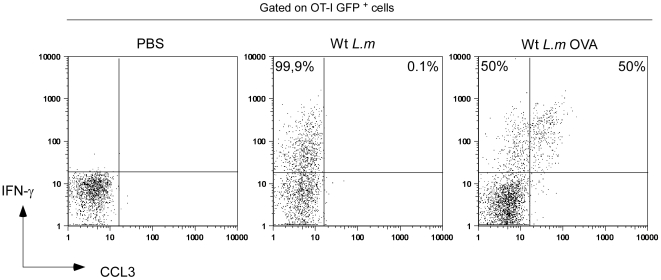
Both *L.m*-specific and non-specific memory CD8^+^ T cells produce IFN-γ, but only TCR-triggered cells secrete CCL3. 200 naïve GFP^+^ OT-I cells were transferred in C57BL/6 mice that were injected i.v. the following day with 0.1×LD_50_ Wt *L.m*-OVA. Thirty days after, recipient mice were injected with 10×LD_50_ Wt *L.m* or Wt *L.m*-OVA. Spleens were harvested 6 hours later, directly stained for IFN-γ and CCL3 expression and analyzed by flow cytometry. The numbers in the quadrants indicate the percentages of IFN-γ ^+^ GFP^+^ OT-I co-secreting or not CCL3.

**Figure 8:**
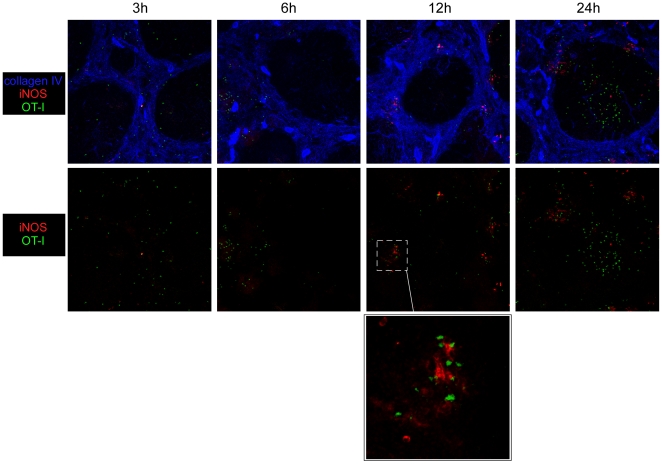
Inflammatory monocytes are activated/recruited to the effector clusters. 200 naïve GFP^+^ OT-I cells were transferred in C57BL/6 mice that were infected i.v. the following day with 0.1×LD_50_ Wt *L.m*-OVA. Thirty days after, recipient mice were injected with 10×LD_50_ Wt *L.m*-OVA. Spleens were harvested 3, 6, 12 and 24 hours later, sectioned, stained for iNOS, collagen IV expression and analyzed by confocal microscopy. Insert shows an enlargement of an effector cluster. Data are representative of 2 different experiments.

We next asked whether such early clustering of memory OT-I cells may be related to the fast control of bacterial growth. We previously demonstrated that reactivated *L.m*-specific CD8^+^ T cells rapidly secrete CCL3, a chemokine that promotes the antimicrobial oxidative burst production in innate immune cells, an effector function required for secondary protection against *L.m* [28]. Others have also shown that memory CD8^+^ T cells rapidly secrete IFN-γ and that such secretion partially contributes to the protective recall response [29], [30]. Finally, several studies have demonstrated that innate cells such as neutrophils and inflammatory monocytes expressing the myeloid marker Ly-6C -also defined as TNF/NO-producing DCs (Tip-DCs)- produce antimicrobial mediators known to be critical for pathogens clearance [31], [32]. Therefore, we characterized the composition of the OT-I clusters induced 6 hours after the challenge infection and looked for the presence of the innate immune cell types and effector functions described above. Spleens sections from Wt *L.m*-OVA challenged mice (6 h) were stained for the expression of B220, CD8, CD4 (Figure 6A); CD11c, *L.m* antigens (Figure 6C) and CD8, IFN-γ (Figure 6D). Attention was focused on the clusters of memory OT-I cells. Data show that these clusters were massive (>100 µm), composed of both CD4^+^ and CD8^+^ T cells (but not B cells) and were usually found around *L.m*-infected CD11c^+^ cells, similar to what was recently described during the secondary challenge infection against *Toxoplasma gondii* in LNs [18]. Most interestingly, we observed both on tissue sections and by flow cytometry an intense IFN-γ secretion by memory OT-I cells in these clusters that we therefore named “effector clusters” (Figure 6D). Although we were unable to detect CCL3 on sections, likely due to a low expression of this chemokine, we did observe CCL3 secretion in memory OT-I cells by flow cytometry. Interestingly, CCL3 expression was always observed in IFN-γ secreting OT-I cells (Figure 7). As IFN-γ secretion by memory OT-I cells was only observed in these effector clusters, we concluded that OT-I cells secreting CCL-3 were also localized in these clusters. To determine if neutrophils were also present there, we took advantage of the GFP-expressing lys-M knock-in mouse in which neutrophils express high amounts of GFP [33]. Massive and local accumulations of neutrophils were indeed found in these clusters (Figure 6B), in agreement with our previous study demonstrating that these cells underwent activation at such early time following the secondary infection [28]. Inflammatory monocytes produce radical oxygen intermediates (ROI) critical for secondary protection against *L.m* infections ([28] and unpublished data). As these intermediates cannot be detected by immunofluorescence, we used the ability of activated inflammatory monocytes to express the inducible nitric oxide synthase (iNOS) to locate these cells on tissue sections [34]. Spleen sections of memory mice challenged for 3, 6, 12 and 24 hours with wt *L.m*-OVA were stained for iNOS and collagen IV expression (Figure 6E and Figure 8). At 6 hours, the peak of the effector cluster formation, we failed to detect iNOS expression in agreement with previous studies [34]. At 12 hours, however, iNOS expression was detected in the OT-I remaining clusters localized in the RP, suggesting that inflammatory monocytes activation and/or recruitment also occurs in these clusters. At 24 hours, when the majority of OT-I cells had relocalized to the WP, we were still able to visualize patches of iNOS-expressing cells in the RP nearby OT-I cells. Thus altogether, these results demonstrate that, very early after a secondary infection, *L.m*-specific memory CD8^+^ T cells aggregate in clusters in the splenic RP where they release effector molecules nearby *L.m*-infected innate immune cells. At the same time and later on, neutrophils as well as inflammatory monocytes are also found in these effector clusters producing antimicrobial mediators such as ROI that are essential for the control of the secondary bacterial burden [28].

### *Both L.m*-specific and non-specific memory CD8^+^ T cells enter effector clusters and produce IFN-γ, but only TCR-triggered cells secrete CCL3

**Figure 9:**
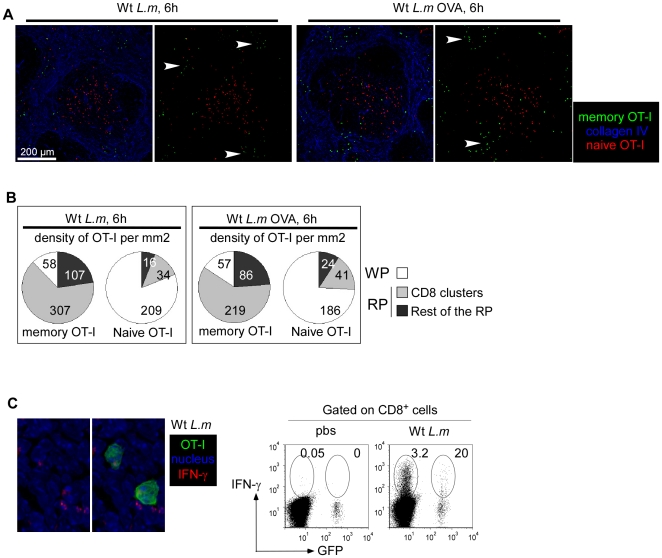
*L.m*-specific and non-specific memory CD8^+^ T cells aggregate in effector clusters and secrete IFN-γ upon *L.m* reinfection. 200 naïve GFP^+^ OT-I cells were transferred in C57BL/6 mice that were injected i.v. the following day with 0.1×LD_50_ Wt *L.m*-OVA.. Thirty days after, recipient mice were injected with 3×10^6^ naïve CMTPX labelled OT-I cells. The following day, mice were injected i.v. with 10×LD_50_ Wt *L.m* or Wt *L.m*-OVA. Spleens were harvested 6 hours later, sectioned, stained for collagen IV (A) or IFN-γ (C) expression and analyzed by confocal microscopy. In C, IFN-γ secretion by endogenous CD8^+^ T cells and memory GFP^+^ OT-I cells was also assessed by flow cytometry. (B) Spleen sections were stained for collagen IV and CD8 expression. The surfaces of 3 regions of interest were drawn: the WP, the RP area containing endogenous CD8 clusters and the rest of the RP. The densities of memory GFP^+^ OT-I and naïve CMTPX OT-I cells per mm2 of each region of interest were calculated and displayed. Data are representative of 2 different experiments.

**Figure 10:**
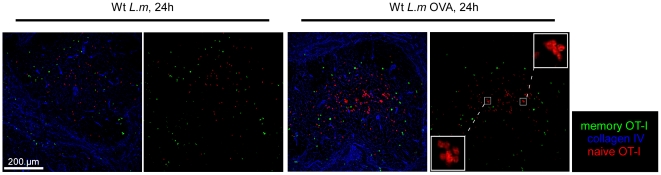
*L.m*-specific naïve CD8 T^+^ cells are reactivated in the WP. 200 naïve GFP^+^ OT-I cells were transferred in C57BL/6 mice that were injected i.v. the following day with 0.1×LD_50_ Wt *L.m*-OVA. One month later, recipient mice were injected i.v. with 3×10^6^ naïve CMTPX labelled OT-I cells. One day after, mice were injected i.v. with 10×LD_50_ Wt *L.m* or Wt *L.m*-OVA. Spleens were harvested 24 hrs later, sectioned, stained for collagen IV expression and analyzed by confocal microscopy. Inserts show the clusterization of naïve OT-I cells in the WP of Wt *L.m*-OVA challenged animals. Data are representative of 2 different experiments.

*In vitro*, memory CD8^+^ T cells are able to rapidly secrete IFN-γ in response to inflammatory cytokines such as IL-12 and IL-18 in absence of any TCR-triggering. Interestingly, Forman et al. have demonstrated that this non-specific secretion contributes to the control of *L.m* growth in the spleen of secondary infected mice [29]. Therefore, we sought to determine if *L.m*-specific and non-specific memory CD8^+^ T cells were equally able to join the effector clusters and express effector functions during secondary *L.m* infection. To this aim, we adoptively transferred 200 GFP^+^ OT-I cells in recipient mice that were injected the day after with 0.1×LD_50_ Wt *L.m*-OVA. One month later, mice were challenged for 6 hours with 10×LD_50_ Wt *L.m* or Wt *L.m*-OVA bacteria and their spleens sectioned and stained for collagen IV expression (Figure 9A). In order to compare if OT-I memory cells were able to join effector clusters in absence of a specific TCR trigger, we also co-stained these sections for CD8 expression and delineated 3 regions of interest: the entire splenic WP, the clusters of endogenous CD8^+^ T cells in the RP and the rest of the RP. Densities of OT-I cells present in these 3 regions were calculated (Figure 9B). Data show that memory OT-I cells were equally capable to join effector clusters and secrete IFN-γ to the same proportion (∼20% of all OT-I cells in both conditions) in mice challenged with Wt *L.m* and Wt *L.m*-OVA, indicating that the capacity of memory CD8^+^ T cells to cluster and provide a rapid response does not require antigen-specific re-activation of the cells (Figure 9A, B and C).Interestingly, we detected CCL3 secretion by memory OT-I cells only in the spleens isolated from animals injected with Wt *L.m*-OVA, suggesting that memory CD8^+^ T cells need antigen-specific stimuli to secrete CCL3 in contrast to IFN-γ (Figure 7). Finally, we also investigated whether naïve OT-I cells were able to join the effector clusters of secondary infected mice or if this capacity was restricted to memory OT-I cells. For this, we used the same experimental setup but adoptively transferred the immunized mice with 3×10^6^ CMTPX-labelled naïve OT-I cells before re-infection. Spleens were harvested 6 hours later, and the densities of memory and naïve OT-I cells were calculated in the 3 regions of interest delineated above (Figure 9A and B). Data show that naïve OT-I cells failed to integrate the effector clusters in both groups of mice. However, as anticipated, naïve OT-I cells aggregated in typical “activation clusters” only in the splenic WP of mice challenged for 24 hours with Wt *L.m*-OVA (Figure 10 and [15]), indicating that naive CD8^+^ T cell activation during a secondary response takes place in the WP yet much after memory CD8^+^ T cells reactivation had occurred in the RP.

## Discussion

Using *L. monocytogenes* as an infection model, we have investigated the behaviour of memory CD8^+^ T cells within the first hours following a secondary bacterial infection. We observed that memory CD8^+^ T cells start to control *L.m* burden as early as 6 hours after the challenge infection. We found that this protection correlated with several properties of these cells including (i) their unique trafficking pattern, (ii) their rapid clustering in the RP upon inflammation driven stimuli and (iii) their local, cluster-localized release of several effector molecules triggered by antigen-independent and dependent signals nearby other innate cell types known to be important for *L.m* clearance.

A simple and obvious question emerges from these observations: what is the purpose of these effector clusters?

First, these clusters may regulate memory CD8^+^ T cells reactivation. At steady state, the majority of memory CD8^+^ T cells traffic in the RP. As we and others observed that memory OT-I cells migrate to the WP 24 hours after the challenge infection in an Ag-dependent fashion (Figure 10, [17]), the only place where these memory OT-I cells may have first encountered their Ag is the RP (though later Ag recognition events in the WP are likely given the relocation of the memory OT-I cells to this zone 24 hrs after the challenge infection). Upon i.v. injection, *L.m* enters the spleen in the marginal zone (MZ) where it is captured by professional phagocytes [14], [15], [16]. Since memory CD8^+^ T cells are activated by CD11c^+^ cells known to rapidly migrate from the RP to the WP, memory CD8^+^ T cells that do not patrol the WP only have a small window of opportunity to encounter their Ag in this location. This requirement correlates with our observation that most memory CD8^+^ T cells are continuously patrolling the blood circulation while avoiding the T cell zones of the SLOs. Of note, the situation may be different for 1/3 of the memory OT-I cells that patrol the WP and therefore may encounter the Ag for the first time in this region.

The observation that memory CD8^+^T cells preferentially patrol the RP may either be dictated by some unknown attracting signal or the consequence of their failure to enter the WP because of their unresponsiveness to CCR7 ligands [35]. Interestingly, CD8^+^ T cells genetically modified to constitutively express CCR7 cannot leave the WP and are impaired in their capacity to efficiently clear a viral infection [36]. For these reasons, we believe that the presence of memory CD8^+^ T cells in the RP results from their inability to enter in the WP and may be important for the rapid release of effector functions upon *L.m* challenge. Along the same line, the preferential trafficking of memory OT-I cells in the bloodstream likely results from their inability to enter the LNs, a phenomenon also observed in CD62L deficient mice [37]. Our observation that memory CD8^+^ T cells are poorly sensitive to the S1P agonist FTY720 further suggests that in addition to their homing properties, the egress of naive and memory CD8^+^ T cells from SLOs are differentially regulated. While we haven't investigated the member(s) of the S1P receptors family known to be affected by FTY720, we can only speculate that S1P1, the master player controlling naive lymphocyte egress from the LNs is not expressed on memory CD8^+^T cells that do not home to LNs. Further experiments would be needed to test this hypothesis.

The biased patrolling of memory CD8^+^ T cells may be crucial for 2 reasons. First, if a *L.m*-specific memory CD8^+^ T cell is wandering for hours in the T cell zone of another SLO when *L.m* arrives in the spleen, this cell will reach the spleen much after pathogen-infected CD11c^+^ cells have left the RP, therefore preventing its reactivation. Second, blood circulating T cells enter the spleen in the MZ, at the very same location than any i.v. injected pathogens [38]. Since memory CD8^+^ T cells traffic in the blood compartment, they indeed have no other choice than meeting the Ag-loaded CD11c^+^ cells in the MZ even at the very beginning of the infection. It is therefore interesting to point out the similarity with the naïve T/DC activation clusters that form nearby HEVs as soon as T cells leave the bloodstream and enter the LN [5]. In both situations, these encounters are dictated by SLO anatomy and aimed to the same goal: increasing the efficacy of the searching process while decreasing its duration.

Second, besides their possible role in memory CD8^+^ T cell reactivation, these effector clusters may also be crucial for controlling *L.m* growth. Since bacteria divide exponentially, controlling their growth at the onset of the infection is essential. Such control may either occur via a direct killing of infected cells and/or by enhancing their microbicidal activities. Memory CD8^+^ T cells can express rapid lytic functions involving perforin/granzyme and Fas/Fas-L dependent pathways to lyse infected cells. However, mice deficient for these molecules are protected during secondary *L.m* infection, indicating that these mediators may not be critical for protection. We have recently demonstrated that CCL3 secreted by memory CD8^+^ T cells is required for the clearance of a secondary *L.m* infection [28]. CCL3 induces a rapid TNF-α secretion by inflammatory monocytes that further promotes the production of ROI in inflammatory monocytes and neutrophils. Indeed, mice unable to produce ROI (phox47^−/−^ mice) do not control a secondary *L.m* infection unless adoptively transferred with Wt neutrophils or inflammatory monocytes, demonstrating that ROI secretion by these cells is required for secondary *L.m* killing. Likewise, IFN-γ secretion by memory CD8^+^ T cells also contributes to the control of a secondary *L.m* infection since IFN-γ^−/−^ mice transferred with Wt but not IFN-γ^−/−^ memory CD8^+^ T cells exhibit much less susceptibility to *L.m* infection [29]. Because *in vitro* studies suggested that IFN-γ prevents bacterial escape from the phagosomes of infected macrophages, it is likely that such indirect mechanism is accounting for this IFN-γ-dependent *L.m* killing *in vivo* [39]. Collectively, all these data support the hypothesis that indirect killing orchestrated by *L.m*-specific memory cells is required for secondary *L.m* protection. In line with this idea, we indeed observed that professional phagocytes transiently aggregate in these effector clusters. Thus, the secretion of IFN-γ and CCL3 by memory CD8^+^ T cells nearby professional phagocytes are likely activating their microbicidal functions, eventually leading to bacteria killing. Of note, the size and composition of these clusters is reminiscent of the granulomas usually observed in slow growing bacterial infections such as *Mycobacterium tuberculosis* or *Mycobacterium bovis*/BCG infections [40], [41]. Since the main function of granulomas is to contain and prevent bacteria dissemination, it is likely that the effector clusters generated in secondary *L.m* infections have a similar purpose. Interestingly, similar to BCG infections in which unspecific effector CD8^+^ T cells enter the granulomas, memory CD8^+^ T cells entry into these clusters does not depend on Ag recognition [42]. Collectively, these results suggest that the capacity to form granuloma-like structure is a feature of effector/memory-but not naïve CD8^+^ T cells- that is independent of their specificity.

Another unexpected finding of our work is that memory OT-I cells were equally able to rapidly secrete IFN-γ in response to Wt *L.m* and Wt *L.m*-OVA challenges. In this regard, IFN-γ secretion by memory CD8^+^ T cells mimics innate immune responses triggered by pathogen-derived “danger signals”. Interestingly, this secretion can be induced by IL-12 and IL-18. Therefore, we can speculate that *L.m*-induced inflammatory stimuli such as IL-12 and/or IL-18 (and others that remain to be defined) activate the bystander secretion of IFN-γ observed in memory CD8^+^ T cells [43], [44]. On the contrary, CCL3 secretion by memory OT-I cells only occurred after wt *L.m*-OVA re-infection, indicating that CCL3, but not IFN-γ secretion, requires an Ag-specific reactivation of the cells. Therefore, the delivery of CCL3 by memory CD8^+^ T is tightly regulated in a conventional “adaptive” manner. These results demonstrate that memory CD8^+^ T cells localized in the same effector clusters are able to secrete various effector molecules according to their reactivating stimuli. If both IFN-γ and CCL3 exhibit a potent pro-inflammatory activity on various cell types, only CCL3 possesses chemotactic properties [45], [46], [47]. Therefore, we can speculate that a tight regulation of CCL3 secretion properly attracts responding cells while a broader IFN-γ secretion creates a local environment unfavorable to bacterial growth as previously suggested by Forman et al. [29].

Finally, while effector clusters contained IFN-γ secreting non-specific memory CD8^+^ T cells, they failed to recruit the *L.m*-specific naive CD8^+^ T cells. Several reasons may account for this observation. It has been estimated that in a mouse, 50–200 naive CD8^+^ T cells are specific for a given MHC/peptide complex [21], [22], [23], [24]. In addition, naïve T cells endlessly patrol the T cell zones of SLO in which they remain several hours. Therefore, it is very unlikely that, if present in the bloodstream at the time of infection, rare *L.m* specific CD8^+^ T cells will home to the spleen rather than to any of the numerous LNs for which they expresses high amounts of the L-selectin homing receptor [37]. Furthermore, upon a primary Wt *L.m*-OVA infection, the progeny of the 200 OT-I cells transferred into C57BL/6 mice represents ∼1% of total splenic CD8^+^ T cells, outnumbering by several logs of magnitude the number of endogenous *L.m*-specific naïve CD8^+^ T cells. Of note, we observed the same phenomenon while studying the endogenous CD8^+^ T cell response in BALB/c mice. Finally, unlike effector and memory CD8^+^ T cells, naïve T cells do not possess any of the intermediate effector functions known to be important for *L.m* clearance. Therefore, these results fit with the inability of these cells to join the effector clusters.

**Figure 11:**
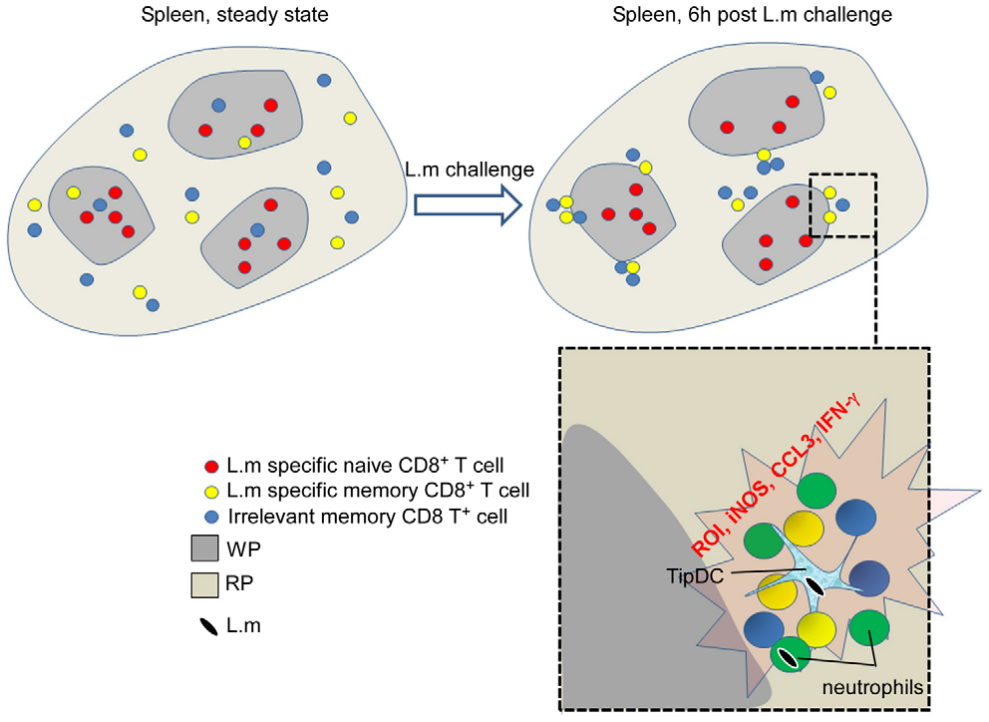
Summary. At the steady state, while splenic *L.m*-specific naive CD8^+^ T cells reside in the WP, *L.m* specific memory cells preferentially traffic in the RP as a result of their increased patrolling of the blood system. Within six hours post *L.m* re-infection, clusters of cells rapidly form around *L.m*-infected cells in the splenic RP of challenged animals. These clusters contain several immune effector cells that express antimicrobial activities such as IFN-γ and CCL3 secreting memory CD8^+^ T cells, neutrophils and Tip-DCs. Importantly, the capacity to join these clusters and secrete IFN-γ is independent of TCR recognition but requires the CD8^+^ T cells to belong to the memory lineage. Because these clusters that involve many antimicrobial effector cells and molecules occur at the exact same time when *L.m* burden starts to be controlled by memory CD8^+^ T cells, we believe that they may be the first site where *L.m* infection is controlled during a recall splenic infection.

Altogether, our results summarized in Figure 11 reveal how memory CD8^+^ T cells unique trafficking properties associated to splenic anatomy result in the formation of transient granulomas-like effector clusters in the RP of secondary infected animals. Our observations provide a spatio-temporal explanation to the subsequent cascade of effector functions that ultimately results in an efficient control of bacterial burden very early after re-infection.

## Materials and Methods

### Mice

BALB/c and C57BL/6 were purchased from Charles River Laboratories (Les Oncins, France). OT-I TCR-transgenic mice [48] were purchased from Taconic (Rockville, MD). C57BL/6 ubiquitin-GFP mice (UBI-GFP/BL6, strain 4353) were purchased from the Jackson Laboratory (Bar Harbor, Maine) while C57BL/6 LysM-EGFP mice were a gift from T. Graf (Albert Einstein College of Medicine, NY). All mice were maintained in the animal facilities of the Centre d'Immunologie de Marseille-Luminy and used between 6 and 12 weeks of age unless specified. Unless specified, groups of mice were composed of 2 animals. All research involving animals has been specifically approved by the ethical committee of the Université de la Méditerranée and the regional committee of the côte d'azur.

### Bacteria, infections and measure of bacterial titers

Wild type and recombinant *L.m* expressing the gene for OVA (Wt *L.m*-OVA) were grown as described previously ([28]). Wt *L.m*-OVA was a kind gift from Dr. Hao Shen (U. Penn, PA, USA). For *L.m* infections, bacteria were grown to a logarithmic phase in broth heart infusion (BHI) medium (Sigma Aldrich) diluted in PBS and injected i.v in the retro-orbital vein. In all experiments, mice were primary immunized with a 0.1×LD_50_ of bacteria (3×10^3^ Wt *L.m* or 10^4^ Wt *L.m*-OVA). *L.m* expressing OVA exhibits a lower LD_50_ than the non-expressing one. Expressing exogenous proteins inside *L.m* usually results in a loss of virulence which is a more general phenomenon observed in infectious agents [49]. Secondary infections were performed 1 month later with 3×10^5^ Wt *L.m* or Wt *L.m*-OVA. To measure bacterial titers in the spleen, organs were harvested and dissociated on metal screens in 10 ml of 0.1% triton X-100 (Sigma Aldrich). Serial dilutions were performed in the same buffer, and 100 µl was plated onto BHI media plates. CFU numbers were counted 24 hours later.

### Adoptive transfers

T cells were purified from the LNs of OT1 TCR-transgenic mice or Wt animals using a pan T cell isolation kit (Miltenyi Biotec, Auburn, California) and stained with either CFSE (2 µM) or CMTPX (5 µM) (Invitrogen, Carlsbad, California) at 37°C for 15 min. The indicated numbers of cells were transferred into host mice by intravenous injection.

### FTY720 and anti-CD8β treatment

When indicated, mice received 100 µg of FTY720 (Cayman Chemical Company, MI, USA) intraperitoneally and were killed 24 hours later. In order to deplete CD8 T cells, mice were injected 100 µg of anti-CD8β depleting Ab (clone H35-17-2) intraperitoneally for three consecutive days.

### Antibodies

ERTR-7 antibody specific for an unknown FRC-secreted molecule was purchased from Acris Antibodies (Hiddenhausen, Germany). RA3-6B2 antibody specific for B220, 17A2 specific for the CD3 complex, XMG1.2 specific for IFN-γ, 11B11 specific for IL-4, RM4-5 specific for CD4, 53−6.7 specific for CD8 were from BD Biosciences Pharmingen (San Diego, CA). Rabbit polyclonal anti-collagen IV antibody was purchased from Abcam (Cambridge, MA). Anti listeria Rabbit serum was purchased from Difco (Lawrence, KS). Rabbit polyclonal anti-NOS-2 antibody was purchased from Santa Cruz Biotechnology (Santa Cruz, CA). Rabbit polyclonal anti-GFP antibody was purchased from Invitrogen (Carlsbad, CA). Goat polyclonal anti-mouse CCL3 antibody was purchased from R&D Systems (MN, USA). These antibodies were visualized by direct coupling to allophycocyanin, pacific-blue, Alexa fluor −488, −568, −647, or through the use of Alexa fluor −488, −568, or −647 coupled secondary antibodies (Invitrogen, Carlsbad, CA). PE-conjugated LLO_91_–_99_/H-2K_d_ tetramers were provided by the NIH (Bethesda, MD).

### Flow cytometry

Cells were stained with the specified antibodies in 100 µl of PBS containing 0.5% BSA (FACS buffer). For intracellular staining, spleens were digested for 20 min in collagenase I (400 U/ml, Invitrogen, Carlsbad, CA) and directly incubated for 20 min on ice with the indicated cell surface marker mAbs, fixed with the FACS buffer for 10 min on ice (*BD* Cytofix/Cytoperm), and permeabilized for 2 min (BD Perm/Wash). Cells were incubated for 20 min on ice in BD Perm/Wash containing anti–IFN-γ (clone XMG1.2) or anti-IL-4 (clone 11B11) Ab as control rat IgG_1_. cells were washed and analyzed on a FACSCalibur cytofluorometer (Becton Dickinson).

### Immunostaining

Spleens were cut in 3 pieces and fixed 1 hr with 8 ml of 2% paraformaldehyde in PBS, washed in phosphate buffer, and dehydrated in 30% sucrose in phosphate buffer for 12 hrs. Spleens were snap frozen in Tissue-Tek® (Sakura Finetek). 20 µm frozen sections were cut and then stained with the indicated antibodies as previously described. Immunofluorescence confocal microscopy was performed using a Leica TCS SP5 confocal microscope. Separate images were collected for each fluorochrome and overlaid to obtain a multicolor image. Final image processing was performed using ImageJ software (National Institutes of Health) and Adobe Photoshop.

### Quantification of the OT-I densities in the WP and RP areas

Immunofluorescence images were segmented into WP and RP areas according to the collagen IV staining. The numbers of pixels of each area as well as the numbers of OT-I were measured using ImageJ software (National Institutes of Health). Finally, densities of OT-I cells per mm2 of WP and RP were calculated. For each condition, a minimum of 20 different fields were counted per spleen, representing an area of interest >3 mm^2^.
